# Visualization of the relationship between metabolism and lung diseases from the perspective of bibliometric analysis: research trends and future prospects

**DOI:** 10.3389/fmed.2024.1443926

**Published:** 2024-11-27

**Authors:** Ming-Yan Wang, Xing-Yu Mo, Meng-Xu Yi, Hong-Yan Lu

**Affiliations:** Department of Pediatrics, The Affiliated Hospital of Jiangsu University, Zhenjiang, China

**Keywords:** cell metabolism, lung diseases, bibliometrics, CiteSpace, VOSviewer

## Abstract

**Background:**

Extensive research has examined the role of metabolism in lung disease development, yet a comprehensive literature review remains absent despite numerous publications.

**Objective:**

This study aims to visualize and assess the advancements in research on metabolism and its role in lung diseases.

**Methods:**

Publications from January 1, 1991, to April 30, 2024, related to lung diseases and metabolism were sourced from the Web of Science Core Collection and analyzed using CiteSpace 6.2.R4, VOSviewer 1.6.19, Bibliometrix, R Studio, and various online tools.

**Results:**

A total of 1,542 studies were collected and processed through these platforms for literature analysis and data visualization. The analysis revealed a sharp increase in annual publications on metabolism and lung diseases, with the United States and China emerging as leading contributors. Current research trends highlight a shift toward investigating metabolic reprogramming of immune cells in the context of lung diseases. Moreover, genes such as TNF, DIF, AKT1, INS, IL-6, CXCL8, IL-1β, TP53, NF-κB1, MTOR, IFNG, TGF-β1, HIF1α, VEGFA, IL-10, NFE2L2, PPARG, AKT, CRP, STAT3, and CD4 have received significant attention in this research domain. Employing a bibliometric approach, this study offers a comprehensive and objective examination of the knowledge landscape, shedding light on the evolving trends in this field. The findings serve as a valuable resource for researchers, offering a clearer perspective on the advancements in metabolism-related lung disease studies.

## 1 Introduction

The relationship between lung function and metabolism is well-established, with energy supporting both fundamental cellular processes and specialized activities, such as airway clearance and surfactant production. This energy expenditure is vital not only for overall cellular functionality but also for specific lung operations. Advances in technology have enabled researchers to ask more complex questions about how individual lung cells adapt to varying health conditions and diseases. These innovations have facilitated the isolation of highly purified cell populations from digested lung samples (1). Additionally, the Seahorse XF analyzer allows for real-time monitoring of cell metabolism, providing both qualitative and quantitative insights into individual metabolites (2). Over the past decade, there has been a surge in research focusing on cell metabolism within the respiratory system.

Cellular metabolism is driven by both anabolic and catabolic pathways, with mitochondria serving as the central hub for most catabolic processes (3, 4). These pathways not only supply energy and biosynthetic intermediates but also regulate the balance between reactive oxygen species (ROS) production and antioxidant activity. Cellular function relies on glucose, fatty acids, and amino acids as metabolic substrates, which, when metabolized, generate intermediates like acetyl-CoA. These intermediates fuel the tricarboxylic acid (TCA) cycle and oxidative phosphorylation (OXPHOS) in mitochondria, leading to ATP production (5, 6). Lung cell metabolism encompasses a variety of catabolic pathways, including glycolysis, the pentose phosphate pathway, OXPHOS, fatty acid oxidation (FAO), and anabolic lipid synthesis (7). The coordinated regulation of these pathways is essential for maintaining proper lung function. While discussions of cellular metabolism often focus on individual pathways, the integration of all metabolic routes is critical for an organism’s survival. The “metabolic theory” of disease posits that alterations in cellular bioenergetics influence not only individual cell behavior but also broader functional and disease processes (8). In patients with chronic lung disease and animal models, metabolic programs indicate that metabolic dysregulation may contribute to disease pathogenesis and progression (9).

Bibliometrics provides a powerful tool for assessing the impact and significance of research, enabling comparisons across countries, institutions, journals, and scholars (10). It also offers a quantitative approach to evaluating research associations and clusters, revealing the dynamic and evolving nature of scientific inquiry (11). Despite the growing popularity of cell metabolism research, bibliometric studies specifically addressing the intersection of metabolism and lung disease remain scarce.

## 2 Methods

### 2.1 Data source and collection strategies

The Web of Science Core Collection (WoSCC) is renowned for its extensive citation indexing and retrieval capabilities, making it one of the most authoritative and comprehensive databases for acquiring, analyzing, and synthesizing academic information within specific professional fields (12). Compared to other databases, WoS offers distinct advantages in bibliometrics, facilitating complex, focused searches while allowing for precise filtering, query refinement, and direct result analysis. Furthermore, WoS provides highly accurate and consistent citation data, free from redundancies.

**Figure 1:**
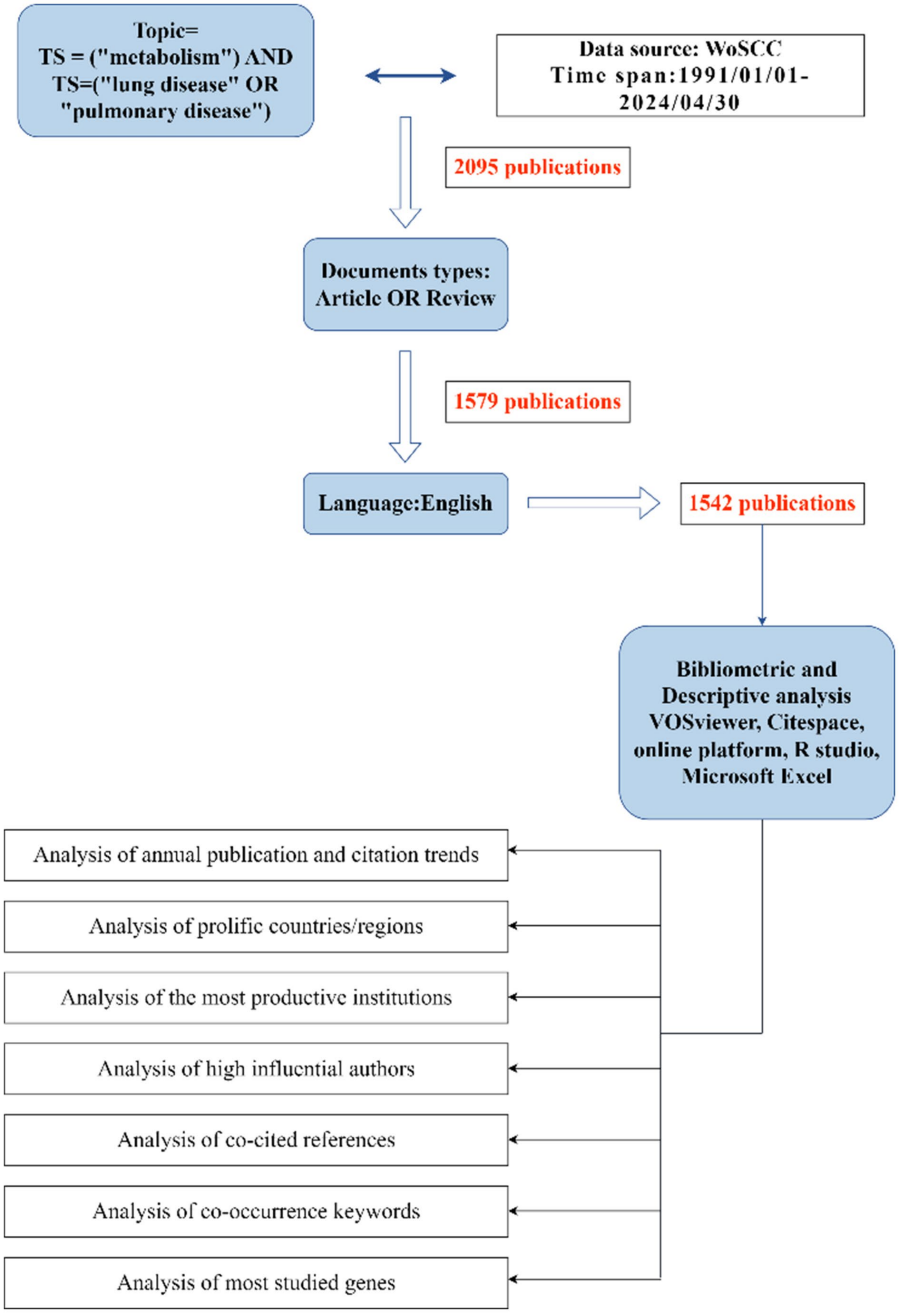
Research process and screening objectives.

In this study, all literature was sourced from the Science Citation Index Expanded (SCI-E, 1991 to present) within the WoS database. To ensure the validity and precision of our results, medical subject terms and research-specific keywords were incorporated into the search parameters. The initial search, TS = (“metabolism”) AND TS = (“lung disease” OR “pulmonary disease”), retrieved 2,095 articles. After excluding 516 non-articles and non-reviews, a final dataset of 1,542 articles was established. The complete retrieval process is illustrated in Figure 1. The final dataset, consisting of 1,542 valid documents, was exported as “complete records and cited references” in a “download_txt” format.

Subsequently, bibliometric indicators such as citation count, country/region, institution, authors, and journals were analyzed. Using data from the 2023 Journal Citation Report^1^, this study obtained metrics such as impact factor (IF), categorical quartiles (Q1, Q2, Q3, and Q4), and other relevant statistics. Additionally, the H-index, a common metric measuring the productivity and citation impact of publications, was utilized based on a descending order of citation frequencies (13).

### 2.2 Bibliometric analysis and data visualization

Three bibliometric tools were employed in this study for scientometric analysis and visualization. CiteSpace version 6.2.R4 (Drexel University, United States), one of the most widely used tools for bibliometric analysis, helps to track the evolution, distribution, and shifts within research fields (14). The study period spans from January 1, 1991, to April 30, 2024. In the visualized network diagrams generated by CiteSpace, nodes represent countries, institutions, or authors, while their size and the colored rings correspond to the frequency of these entities and their respective years.

VOSviewer 1.6.19 (Leiden University, Netherlands) enables deep literature mining (15) by extracting key parameters from vast volumes of scientific publications, creating co-citation and co-occurrence networks for visualization. In this study, VOSviewer was used to generate visual network diagrams covering institutional analysis, author collaboration, journal co-citation, and keyword co-occurrence. Each node in the VOSviewer graph is represented by a labeled circle, with larger circles signifying higher co-occurrence frequencies. The length and thickness of connecting lines between nodes reflect the strength and correlation of their relationships. Additionally, VOSviewer provides three distinct types of network maps: network visualization, overlay visualization, and density visualization.

An online bibliometric analysis platform^2^ was also utilized to examine the annual publication trends and the collaborative intensity between countries (16).

Microsoft Excel 2019 (Microsoft Corporation, Redmond, WA, United States) and GraphPad Prism version 8.0 (GraphPad Software, La Jolla, United States) were used to create visual representations of literature type distribution, publication trends, and the total number of documents published by the top ten countries/regions in this field.

Furthermore, protein–protein interaction (PPI) networks were constructed using the online platform GeneMANIA^3^, while functional enrichment analyses of the most frequently studied genes were performed using R Studio.

## 3 Results

### 3.1 Publication statistics of previous articles

**Figure 2:**
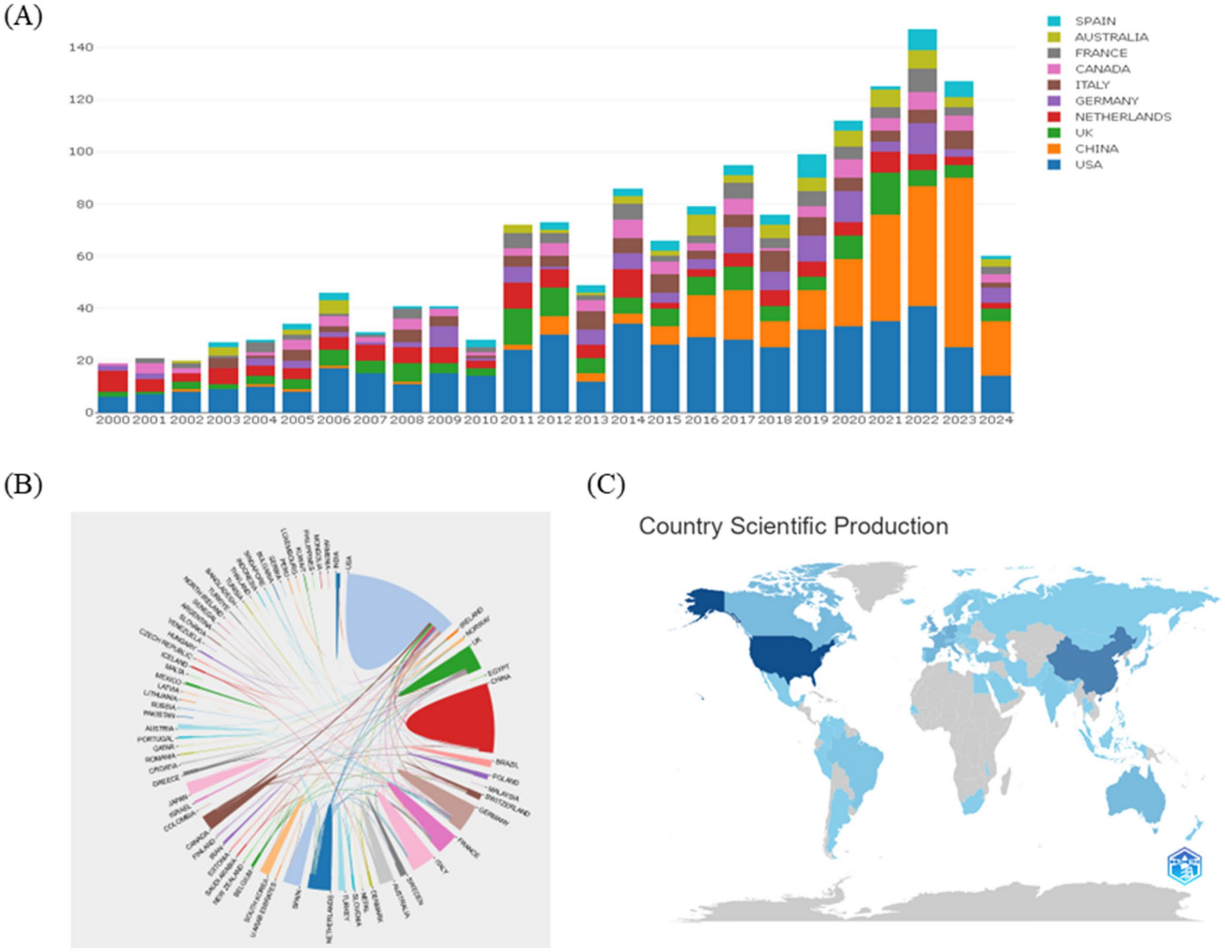
Annual number of publications of articles on lung diseases and metabolism, 2000–2024. **(A)** To analyze the annual growth trend of the top ten countries in the number of articles published on the online analysis platform of metrology on lung diseases and metabolism from 2000 to 2024. **(B)** Analysis of international cooperation between different countries. **(C)** Visualization analysis of country scientific production.

The number of published articles in a scientific field offers valuable insight into its development. In this study, a total of 2,095 articles were retrieved, of which 1,579 (86.03%) were original research papers, and 516 (13.97%) were reviews, with 1,542 articles written in English. Online bibliometric platforms were used to determine the actual annual publication count in this field since 2000 (Figure 2A). The histogram illustrates a steady rise in the number of published articles, reflecting the growing exploration of how metabolism influences disease, especially in modern medicine. This surge highlights increasing attention to the metabolic mechanisms underlying lung diseases. Recent studies have progressively unveiled the pathogenesis and metabolic targets of lung diseases.

To identify the countries/regions contributing significantly to this field, the number of articles published by each country/region was analyzed. The top 10 countries/regions in terms of publication volume over the past 20 years are shown in Figure 2A. The United States is regarded as a leader in this field, with consistent publication growth. Since 2016, China, the United Kingdom, and Australia have also emerged as major contributors, with China showing rapid publication growth since 2018. There are strong expectations for continued growth in Chinese publications. The collaborative networks between different countries are illustrated in Figure 2B, where the thickness of the lines indicates the strength of cooperation between nations. Currently, the United States has strong collaborative ties with China, Canada, and the Netherlands. During this period, major research themes have emerged, including the role of cellular metabolism and metabolic pathways in the progression of lung diseases, and how metabolic reprogramming affects immune cell function. The rise of these topics underscores increasing academic interest in the critical role of metabolism in lung disease.

Overall, strengthening these international collaborations remains essential for advancing the field. Visualization of scientific output by country (Figure 2C) reveals that China and the United States are the top producers of articles. According to Table 1, the United States ranks first, followed by China in second place and Germany in third.

**Table 1:** Top 10 most productive countries/regions in the field of pulmonary metabolism.

Rank	Country/Region	Total citations	Average citations
1	USA	14,405	35.8
2	CHINA	3,226	11.9
3	GERMANY	2,957	46.2
4	UK	3,846	44.7
5	FRANCE	1,123	22
6	NETHERLANDS	205	29.3
7	ITALY	1813	24.2
8	SPAIN	1,194	28.4
9	CANADA	1,406	25.6
10	AUSTRALIA	1,129	25.7

### 3.2 Country and institutional collaboration analysis

**Figure 3:**
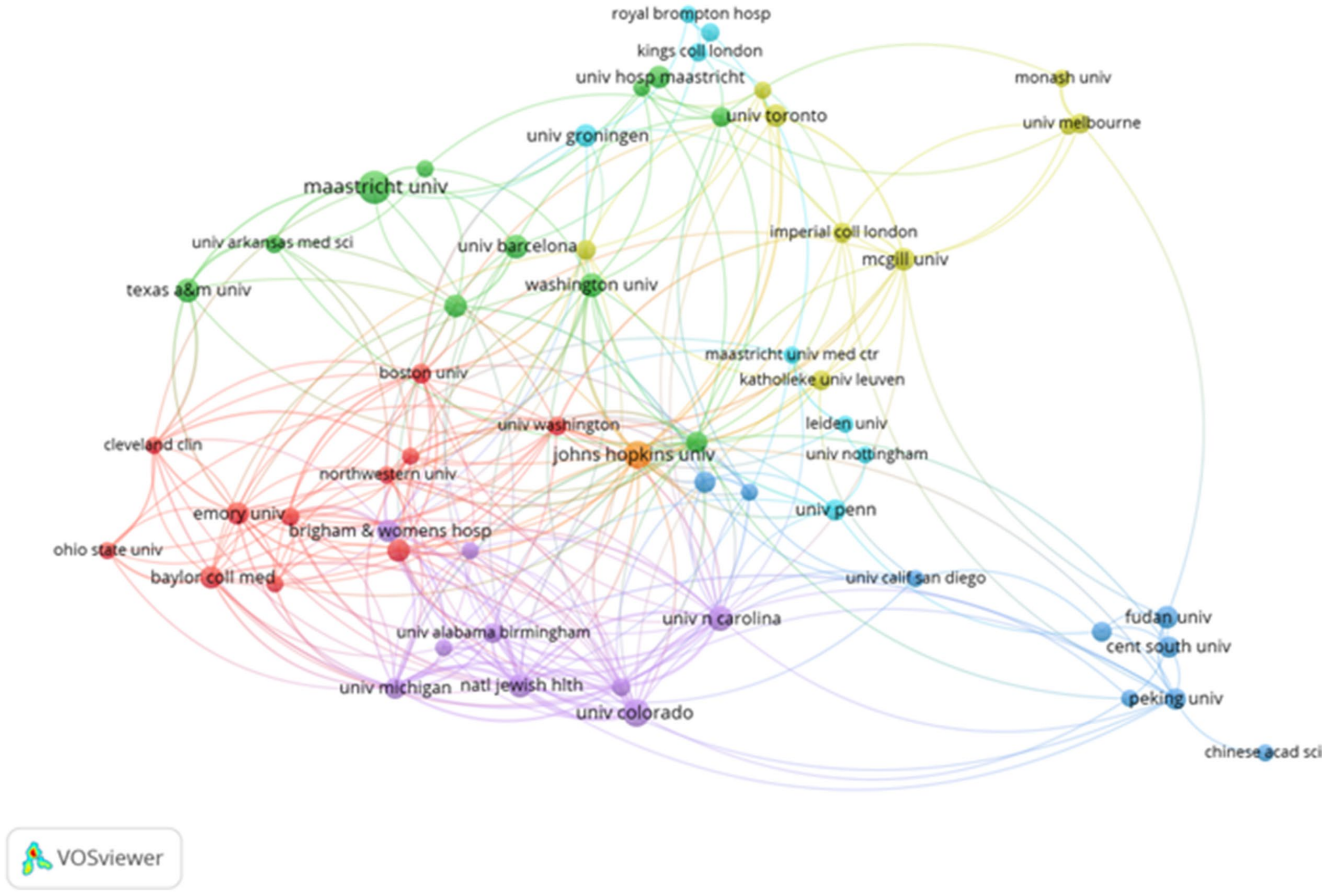
Collaborative visualization maps generated by VOSviewer for the top 58 institutions in terms of publication volume.

Using CiteSpace, a collaboration network involving 70 countries and 1,106 donor agencies was revealed (Figure 3). Table 2 highlights the top 10 institutions with the highest publication output in the field of metabolism and lung diseases. The University of California system ranks first with 78 publications (7.05%), followed by Maastricht University (74, 6.69%), Harvard University (70, 6.33%), Maastricht University Medical Centre (68, 6.15%), Université Paris Cité (66, 5.97%), and the Institut National de la Santé et de la Recherche Médicale (64, 5.79%). The United States maintains a dominant position in publication volume, further consolidating its academic influence in this field. China has established collaborative publications with several countries, including the United States, the United Kingdom, Singapore, Japan, Canada, and Australia. These partnerships focus on shared research areas. The primary research topics in the United States, Italy, and China include genome-wide association studies (GWAS) for lung function and chronic obstructive pulmonary disease (COPD), identifying novel loci and potential druggable targets. Their work emphasizes targets in the inositol phosphate metabolism pathway and CHRM3, aiming to highlight drugs and compounds in development for COPD and asthma, as well as potential drug repositioning opportunities from other clinical fields. Close cooperation between these countries suggests that metabolism pathways and molecular targets in lung diseases are common research themes. Developed countries, leveraging their robust economic and technological resources, are particularly well-positioned to address these research challenges effectively.

**Table 2:** Top 10 institutions performing studies on pulmonary metabolism.

Rank	Institution	Country/Region	Quantity
1	UNIVERSITY OF CALIFORNIA SYSTEM	USA	78
2	MAASTRICHT UNIVERSITY	Netherlands	74
3	HARVARD UNIVERSITY	USA	70
4	MAASTRICHT UNIVERSITY MEDICAL CENTRE (MUMC)	Netherlands	68
5	UNIVERSITE PARIS CITE	France	66
6	INSTITUT NATIONAL DE LA SANTE ET DE LA RECHERCHE MEDICALE (INSERM)	France	64
7	UNIVERSITY OF COLORADO ANSCHUTZ MEDICAL CAMPUS	USA	49
8	UNIVERSITY SYSTEM OF OHIO	USA	56
9	ASSISTANCE PUBLIQUE HOPITAUX PARIS (APHP)	France	50
10	JOHNSHOPKINS UNIVERSITY	USA	50

### 3.3 Author contribution analysis

The number of publications by an author reflects their scholarly activity and contributions to their respective research fields. As indicated in Table 3, SCHOLS AMWJ is the most prolific author with 47 publications, followed by WOUTERS EFM (35 publications) and DEUTZ NEP (29 publications). The H-index, a metric for assessing the impact of an author, journal, or country, represents the number h of publications that have been cited at least h times (13).

**Table 3:** Top 10 authors who publish pulmonary metabolism studies.

Rank	Author	Counts	Citations	H-index
1	SCHOLS AMWJ	47	251	25
2	WOUTERS EFM	35	237	24
3	DEUTZ NEP	29	179	16
4	ENGELEN MPKJ	29	189	17
5	GOSKER HR	21	61	16
6	WANG Y	17	8	7
7	LI Y	16	15	10
8	LI JS	13	15	7
9	RUTTEN EPA	13	4	10
10	WANG J	13	13	7

**Figure 4:**
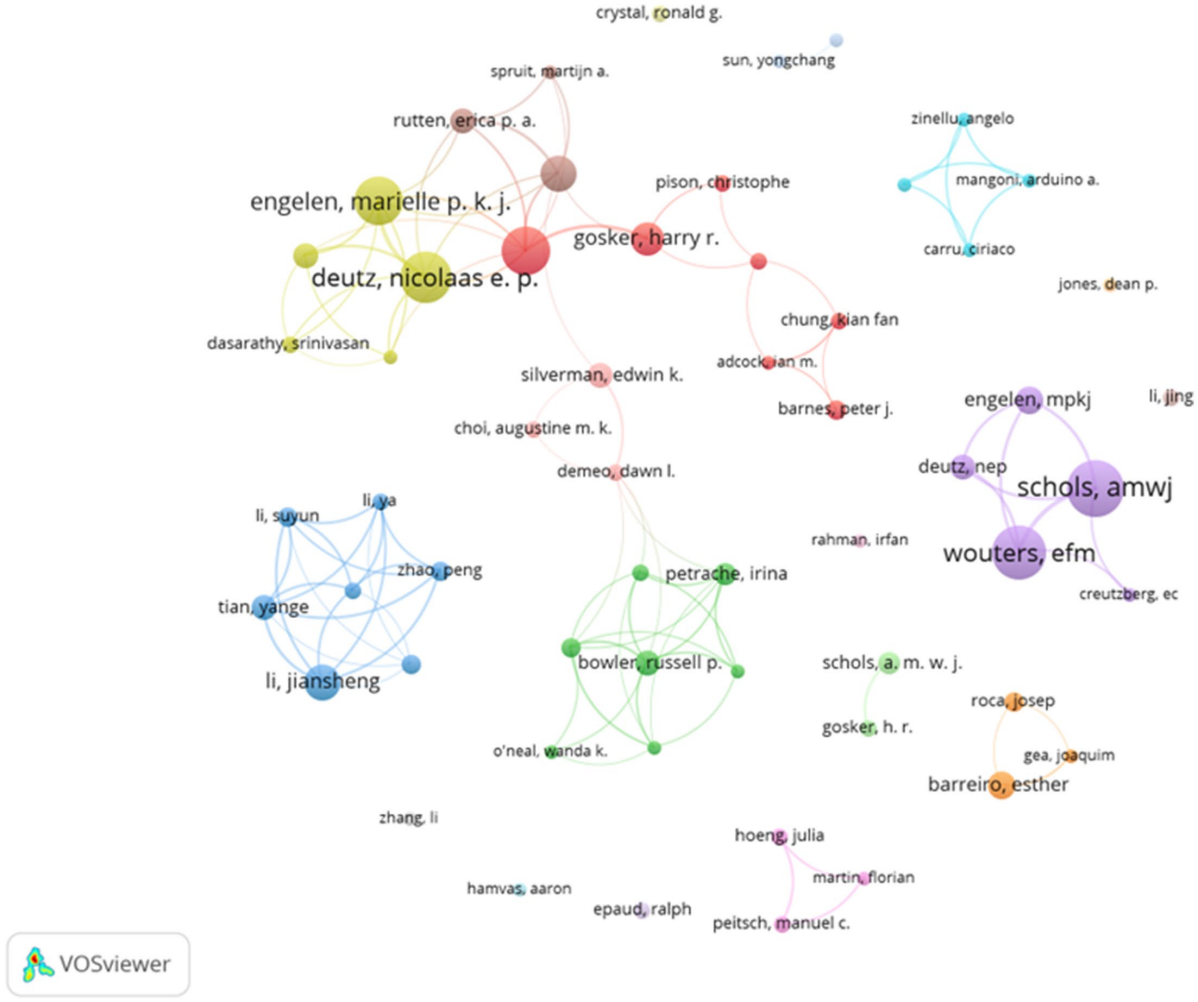
Network map of the authors’ collaborative analysis of pulmonary disease and metabolism 1991–2024.

Each author brings unique expertise to various research topics, and cross-collaborations facilitate greater interaction and quantitative output in the field. Analyzing the collaboration between authors and co-authors provides valuable insights into existing partnerships, which can help foster better communication and encourage the development of collaborative research themes. As visualized in Figure 4, the collaboration map generated by VOSviewer highlights several concentrated research groups, each connected by one or two core authors with significant contributions, such as SCHOLS AMWJ, WOUTERS EFM, DEUTZ NEP, and ENGELEN MPKJ. These authors have notably collaborated on studies examining the metabolic profile of patients with COPD and the effects of glutamate, glutamine, and polyunsaturated fatty acid intake (17, 18). These collaborations, often centered around foundational research, emphasize the need for improved communication and stronger connections between research groups. CiteSpace was also employed to identify mutual collaborations among authors, yielding results consistent with those from VOSviewer.

### 3.4 Analysis of co-citation references

**Figure 5:**
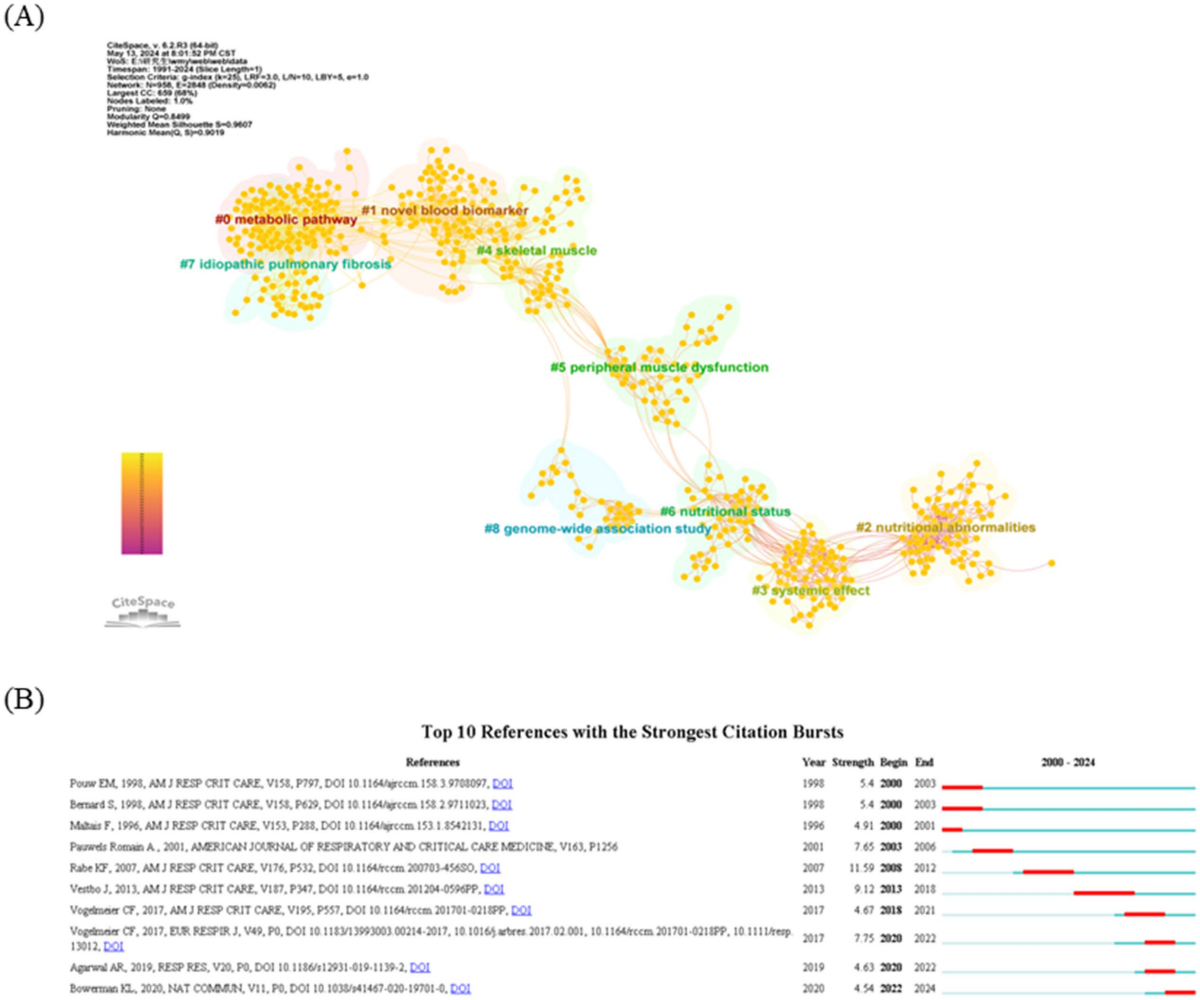
Analysis of reference co-citation and burst references. **(A)** Visualization of the literature co-citation analysis network constructed by CiteSpace. **(B)** Top 10 articles with a spike in citations. The red segments correspond to the start and end years of the outbreak duration.

In addition to author collaboration, reference co-citation analysis is a valuable tool for evaluating how frequently references are cited together, revealing their interconnections and identifying influential studies and key contributors. Using CiteSpace, co-citation network diagrams were created for the period from 1991 to 2024, showcasing interrelated references in an abbreviated format. This analysis produced a visualization of the relationships between co-cited references (Figure 5A). The clustering function in CiteSpace aggregated the network into several distinct clusters, with the eight largest clusters identified as follows: metabolic pathway (cluster #0), novel blood marker (cluster #1), nutritional abnormalities (cluster #2), systemic effect (cluster #3), skeletal muscle (cluster #4), peripheral muscle dysfunction (cluster #5), nutritional status (cluster #6), idiopathic pulmonary fibrosis (cluster #7), and genome-wide association study (cluster #8). Figure 5B highlights the characteristics of the top 10 most co-cited references in the field of metabolism and lung disease, including seminal works by Pouw et al. (19), Bemard et al. (20), Maltais et al. (21). Additionally, the top five references with the strongest citation bursts in this field, listed in Table 4, provide a valuable reference point for future research directions.

**Table 4:** Top 5 most frequently cited papers in the field of metabolism and lung disease.

Times cited	Title	Year	Journal name	JCR	IF	Main conclusions
23	Plasma and muscle amino acid levels in relation to resting energy expenditure and inflammation in stable COPD	1998	AM J Respir Crit Care Med	Q1	24.7	There are different levels of AA in plasma and muscle in patients with COPD
156	Peripheral muscle weakness in patients with COPD	1998	AM J Respir Crit Care Med	Q1	24.7	The distribution of peripheral muscle weakness and the correlation between quadriceps strength and the degree of airflow obstruction in patients with COPD.
77	Oxidative capacity of the skeletal muscle and lactic acid kinetics during exercise in normal subjects and in patients with COPD	1996	AM J Respir Crit Care Med	Q1	24.7	Oxidase activity was significantly lower in the COPD group than in the control group, and lactate increased in the COPD group
24	Systemic Immuno-metabolic alterations in COPD	2019	Respiratory Research	Q1	5.8	Studies have shown that the metabolism of systemic immune cells in COPD patients is impaired, and the ability to metabolize carbohydrates or fatty acids is reduced. To our knowledge, this is the first study to demonstrate these metabolic changes in healthy smokers and patients with COPD.
134	Disease-associated gut microbiome and metabolome changes in patients with COPD	2020	Nature Communications	Q1	16.6	The authors compared the feces of COPD patients and healthy controls to find specific gut bacteria and metabolites associated with active disease, thus hinting at the potential role of the gut microbiome in COPD.

### 3.5 Analysis of keywords co-occurrence and burst keywords

**Figure 6:**
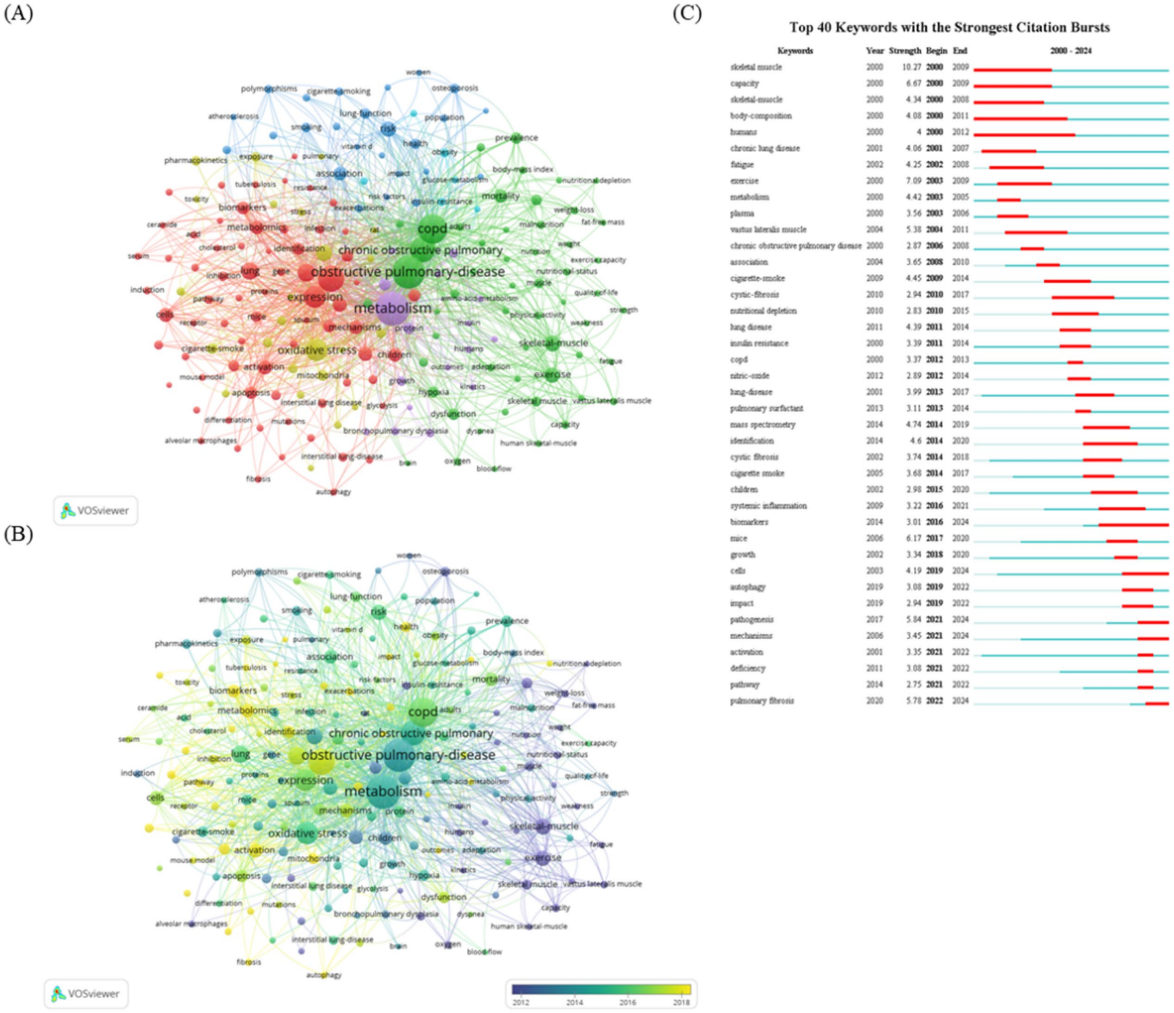
Analysis of co-occurrence and burst keywords. **(A)** VOSviewer keyword visualization map. Keywords are represented by labeled circles. The size of the circles and labels is proportional to the keyword frequency, and circles of the same color belong to the same cluster. Visualization of keyword co-occurrence analysis density. The heatmap visually represents the frequency of keywords by using a range of color shades. Intense red shading indicates active study areas where keywords occur more frequently, while cooler yellow shading indicates inactive areas where keywords occur less frequently. **(B)** Visualization of keyword co-occurrence analysis density. The heatmap visually represents the temporal changes in the occurrence of keywords by using a series of colors. **(C)** Top 25 most frequently used keywords in the research field.

A total of 1,542 articles in this field were analyzed using online tools to identify keywords that appeared at least 30 times. After removing irrelevant terms and merging synonyms, 166 distinct keywords were identified. VOSviewer was employed to examine keyword co-occurrences. As shown in Figure 6A, the keyword density visualization highlights areas of high-frequency occurrence in red, while yellow indicates areas with less frequent keyword use. Figure 6B provides a heatmap, depicting the temporal evolution of keyword usage through varying color intensities. In Figure 6C, the 25 most commonly used keywords in metabolism and lung disease research are displayed. High-frequency terms such as “skeletal muscle,” “chronic lung disease,” “COPD,” “bronchopulmonary dysplasia (BPD),” “biomarkers,” “autophagy,” “mechanisms,” and “pathway” represent key research areas in this field. Research hotspots were identified through keyword biclustering, which grouped these high-frequency terms into clusters, indicating the dominant research directions in metabolism and lung disease studies.

### 3.6 Analysis of hot spot genes

**Figure 7:**
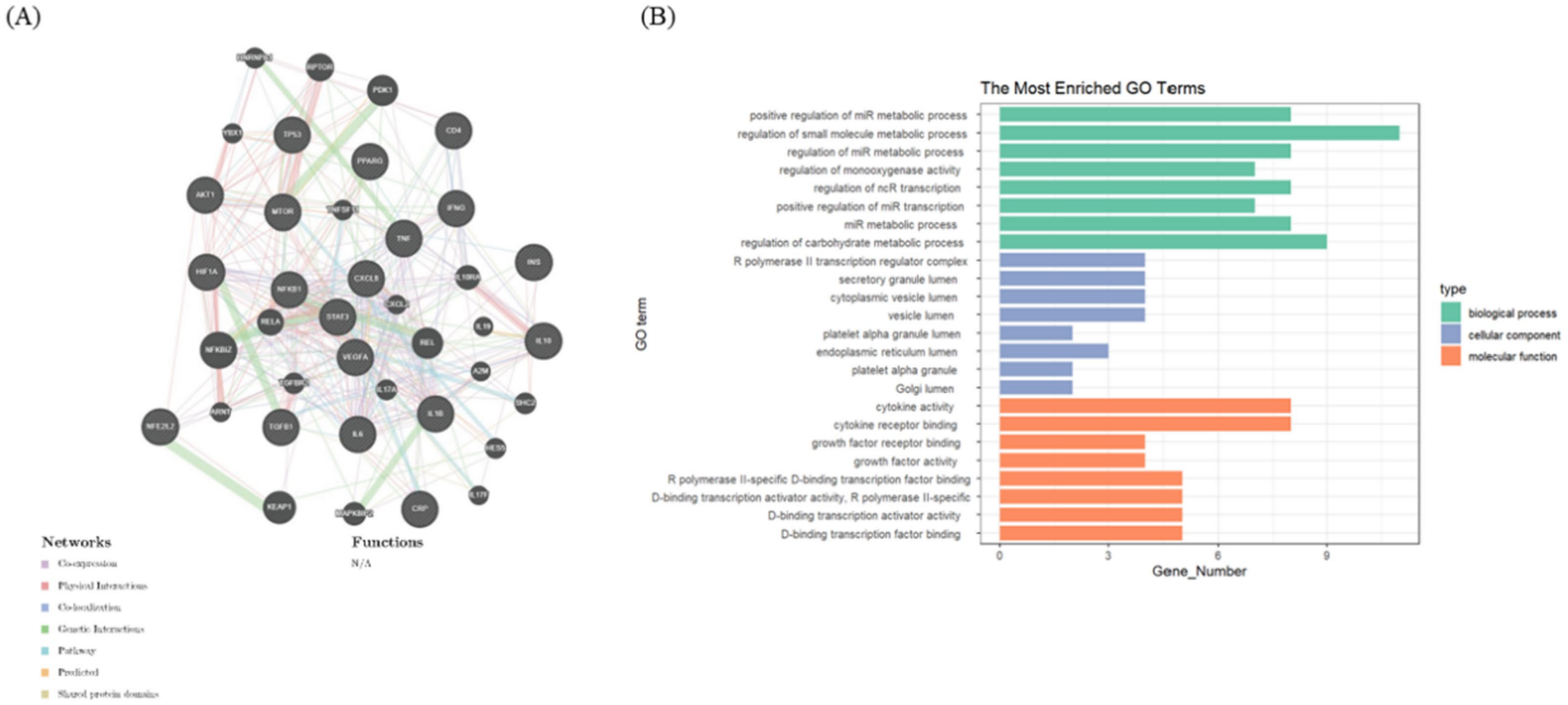
Analysis of hot spot genes. **(A)** The top 21 most studied genes at the intersection of lung disease and metabolism, using online tools to construct gene interaction networks. **(B)** Bar graphs show the GO enrichment analysis of these top related genes.

In addition, a hot spot gene analysis was conducted using online data platforms to identify the most widely studied genes in metabolism and lung diseases. As depicted in Figure 7A, 21 genes, including TNF, DIF, AKT1, INS, IL-6, CXCL8, IL-1β, TP53, NF-κB1, MTOR, IFNG, TGF-β1, HIF1α, VEGFA, IL10, NFE2L2, PPARG, AKT, CRP, STAT3, and CD4, were the most extensively researched in this cross-disciplinary area. To further explore molecular interactions, an interaction network of these genes in lung disease was generated using online tools (Figure 7A). Gene Ontology (GO) enrichment analysis was also conducted on these 21 genes, with the results visualized as bar graphs. Figure 7B reveals that the genes are primarily associated with the regulation of small molecule metabolic processes and cytokine activity.

## 4 Discussion

### 4.1 Summary of the main findings

A comprehensive analysis of articles related to metabolism and lung disease from 1991 to 2024 was conducted using the WoS database, generating various types of bibliometric maps. Researchers employed VOSviewer, CiteSpace, and Bibliometrix to perform both quantitative and qualitative analyses, revealing a strong correlation between metabolism and the progression of lung disease. The study highlights how long-standing influences have shaped the research networks in this field, providing researchers with access to key papers, leading journals, esteemed authors, and emerging trends. The United States has been at the forefront of research efforts, consistently contributing to and fostering international collaboration. The state of scientific research within a country often reflects its overall strength, and between 2015 and 2023, China—a developing nation—has demonstrated significant growth in the number of publications within this domain. At the institutional level, the University of California emerged as the most prominent and highly cited institution. The top authors across the entire study period were identified as SCHOLS AMWJ, WOUTERS EFM, and DEUTZ NEP, while the most frequently cited journals were the American Journal of Respiratory and Critical Care Medicine, the European Respiratory Journal, and Chest. The analysis of the co-citation reference network from 1990 to 2024 uncovered interconnected relationships among 18 distinct research cohorts, shedding light on evolving trends in metabolic processes related to lung disease. These findings provide valuable insights into future research directions and potential collaborative opportunities. Additionally, the expanding body of work on immune metabolism underscores the critical role of metabolic intermediates in regulating immune cell function. This suggests that targeting immune cell metabolism to modify their functional phenotype could offer promising avenues for therapeutic interventions in lung diseases.

### 4.2 Hotspots and frontiers

Emerging research increasingly supports the critical role of metabolism in regulating cellular function, particularly in the context of lung diseases. Metabolic alterations can influence not only individual cell behavior but also contribute to organ dysfunction and the onset of various diseases. A keyword commonality analysis identified that most studies in this field have focused on COPD, BPD, and metabolic pathways, including glycolysis, glucose metabolism, amino acid metabolism, and mitochondrial function.

#### 4.2.1 Alterations of metabolism in COPD

COPD ranks among the top three causes of global mortality, posing a significant public health challenge (22). Cigarette smoke (CS) primarily targets epithelial cells, resulting in marked structural and functional changes in mitochondria, as observed in both mouse models and patients with COPD (23). These alterations include a reduced number of cristae per mitochondrion, enlargement of the organelles, and decreased mitochondrial membrane potential and oxygen consumption rates. Mitochondrial dysfunction in lung epithelium is further linked to the upregulation of metabolic pathways such as glycolysis, *β*-oxidation, and autophagy, underscoring the interconnectedness of these cellular processes.

Recent studies have highlighted a relationship between ciliary dysfunction and decreased cellular energy production in patients with COPD. Metabolic dysregulation has been identified in these patients, with CS exposure leading to impaired glycolysis in type II alveolar cells (24). Furthermore, disruptions in lipid and amino acid metabolism have been noted in both human patients with COPD and mice with CS-induced emphysema (25, 26). Metabolomic analyses of airway basal stem cells from long-term smokers revealed reduced levels of metabolites and cofactors compared to healthy controls. Consequently, metabolite measurements are emerging as potential biomarkers for COPD.

#### 4.2.2 Alterations of metabolism in BPD

BPD is characterized by arrested alveolar development, leading to pulmonary insufficiency in preterm infants (27). High oxygen exposure is considered a major contributor to this condition (27), as supplemental oxygen can interfere with normal lung and pulmonary microvessel growth and development (28). Although many individuals with BPD can eventually be weaned off oxygen therapy, lingering complications such as impaired lung function and cardiovascular issues often persist into adolescence and adulthood (29, 30). Recent research has also highlighted abnormalities in metabolic regulation, including dysregulation of glucose, lipid, and amino acid metabolism. Hyperoxia has been shown to influence the onset and progression of neonatal diseases induced by excessive oxygen levels (31). Previous studies have identified abnormal and enlarged mitochondria with distorted cristae in the lung cells of animals exposed to prolonged hyperoxia. This mitochondrial impairment in lung tissue is recognized as a harmful consequence of extended oxygen therapy (32). Hyperoxia has been linked to cellular senescence and increased glycolytic activity in cultured lung epithelial cells (32, 33), and clinical studies have confirmed elevated glycolysis in individuals exposed to hyperoxic conditions (34).

In a study by Das et al., urine metabolomics of infants diagnosed with BPD at birth revealed elevated lactate levels and reduced gluconic acid levels (35, 36). Hyperoxia was also found to enhance glycolysis and the pentose phosphate pathway (PPP) in endothelial cells of neonatal mice (37). Further investigations into glucose metabolism, both *in vivo* and *in vitro*, revealed that hyperoxia diminished glycolytic capacity, glycolytic reserve, and OXPHOS in MLE-12 cells, while also impairing complexes I and II in isolated mouse lung mitochondria, leading to reduced energy production (38).

Additionally, Ratner et al. documented a reduction in complex I levels in a mouse model exposed to hyperoxia (32). Studies on neonatal mice have shown that hyperoxia alters lung lipids, increasing levels of sphingomyelin, glycerophosphatidylcholine, and glycerolipid species, which may result in alveolar simplification and hinder vascular development (39). Similarly, Frano et al. reported changes in umbilical cord blood lipid levels in infants with BPD, suggesting that disruptions in lipid metabolism may originate during fetal development (40). Carraro et al. further found that adolescents diagnosed with BPD exhibit altered complex lipid profiles in exhaled breath condensates, indicating the possibility of persistent metabolic abnormalities beyond infancy (41). Moreover, Ye et al. observed disturbances in amino acid metabolism in infants with BPD, noting the upregulation of phenylalanine and methionine, along with the downregulation of citrulline, glutamate, alanine, and tyrosine in early blood samples (42).

Piersigilli et al. identified elevated levels of glutamate, histidine, citrulline, asparagine, glycine, and isoleucine in tracheal aspirates from individuals with BPD (43). Additionally, animal models exposed to hyperoxia exhibited decreased levels of L-citrulline and L-arginine in the blood (44). Taurine was also reduced in tracheal aspirates and urine samples from infants with BPD, suggesting its potential as a novel biomarker for the condition (36). The growing use of urine metabolomics as a research tool is due to its non-invasive nature and practicality (36). Variations in study findings may stem from differences in sample size, source, and underlying neonatal conditions.

Oxygen therapy is frequently employed to support neonates’ respiratory function. However, this treatment presents a dual challenge: providing sufficient oxygen to tissues without inducing toxicity or oxidative stress. Excessive oxygen therapy can lead to multisystemic complications in neonates. Unfortunately, the ideal oxygen saturation level for neonates and the long-term safety of oxygen therapy remain unclear, requiring further investigation. Recent studies have shown that hyperoxia induces metabolic reprogramming in neonates, resulting in metabolic dysregulation across various organs, affecting glucose, lipid, and amino acid metabolism. Metabolite abnormalities could serve as predictive biomarkers for disease onset and as potential therapeutic targets, offering promising avenues for future research (43). While the exact mechanisms behind hyperoxia-induced metabolic reprogramming remain incompletely understood, interactions between mitochondria and the lungs, brain, and intestines appear critical in this process. Further research is needed to unravel the metabolic pathway alterations in response to hyperoxia, with the goal of developing preventive and therapeutic strategies for hyperoxia-related neonatal disorders.

#### 4.2.3 Alterations of metabolism in other lung diseases

In patients with BPD, the proliferation and remodeling of pulmonary artery smooth muscle cells, triggering PAH. Changes in cellular metabolic pathways are thought to be critical drivers in this process. Specifically, the upregulation of glycolysis enables cells to generate energy in hypoxic environments, promoting cell proliferation and survival. Furthermore, the inhibition of FAO can disrupt energy balance, exacerbating the pathological state of PAH. Interventions targeting these metabolic pathways offer a new approach for treating PAH.

Metabolic abnormalities have been observed in animal models of PAH. Key metabolic hallmarks of PAH include a shift toward glycolysis, increased glutamine utilization, one-carbon metabolism, and decreased FAO. Elevated ROS production and alterations in TCA cycle intermediates play pivotal roles in linking the vasoconstrictor phenotype of PAH to these metabolic abnormalities.

Cancer cells meet their needs for rapid proliferation and survival by altering their metabolic pathways, and these alterations are often referred to as “tumor metabolic reprogramming” (45). Lung cancer cells preferentially obtain energy through glycolysis, which mainly produces lactate even under aerobic conditions (46). Lung cancer cells increase glucose uptake and activate enzymes related to glycolysis, increase fatty acid synthesis to meet the membrane structural demand of rapid proliferation and cholesterol synthesis and uptake are increased, which is associated with tumor invasiveness and drug resistance (47). Metabolic reprogramming of lung cancer cells often leads to increased oxidative stress, so they need to protect themselves by enhancing antioxidant capacity (48). Glutathione and NADPH are important molecules in maintaining the antioxidant system (49). Lung cancer cells will increase the production of NADPH through metabolic pathways (such as the PPP) to maintain redox balance (50).

Idiopathic pulmonary fibrosis (IPH) is a progressive and fatal interstitial lung disease, and its metabolic changes play an important role in the occurrence and development of the disease (51). In IPF, lung fibroblasts undergo metabolic reprogramming, manifested by increased glycolysis and decreased mitochondrial OXPHOS (52). This metabolic change is similar to the “Warburg effect,” in which activated fibroblasts and myofibroblasts become more dependent on glutamine, which provides energy and biosynthetic intermediates for these cells, and lipid metabolism is also significantly altered. There is a significant increase in oxidative stress in patients with IPF, which is closely related to metabolic reprogramming and dysfunction of antioxidant system (53). Excessive ROS not only damages lung epithelial cells, but also promotes fibroblast activation and excessive matrix deposition (54). Studies have shown that the metabolism of certain amino acids is significantly altered in lung tissues of IPF patients (55). The chronic inflammatory response in IPF is closely related to metabolic reprogramming, especially the metabolic changes of immune cells such as macrophages and T cells (56, 57). The imbalance of metabolic regulation may promote the activation of M2 macrophages, thereby aggravating fibrosis.

Metabolomics studies have shown that lung energy metabolism is significantly affected in patients with asthma. Several studies have found significant changes in amino acid and lipid metabolism in patients with asthma (58). Lipid metabolites play an important role in the pathogenesis of asthma and are also the research direction and hot spot at present. In the development of asthma, lipid molecules are involved in the complex regulation of various cellular functions, such as antigen presentation by dendritic cells (DCs), T cells, B cells and mast cells and other cell types, suggested that these metabolites may play a role in the immune and inflammatory response of asthma. Studies have found that glycolysis, oxidative stress fatty acid and amino acid metabolism of different immune cells are abnormally enhanced, which leads to the imbalance of innate and adaptive immune responses in the pathogenesis of asthma (59). By targeting these metabolic pathways, new therapeutic strategies are expected to be developed.

#### 4.2.4 Immunometabolism in lung disease

Metabolic reprogramming is a critical cellular adaptation to various environmental and cellular stressors. While immune cells share metabolic pathways with other cell types, they exhibit unique metabolic shifts during both innate and adaptive immune responses. To meet the energy and biosynthetic demands required for immune activation, immune cells adjust enzyme activity and nutrient uptake (60, 61). The field of immunometabolism, which examines metabolic processes within immune cells, has existed for over half a century, but it is only in the last decade that attention has shifted toward understanding the metabolic reprogramming involved in immune cell differentiation and activation. Immunometabolism encompasses several metabolic pathways and intermediates, including glycolysis, the Krebs cycle, the pentose phosphate pathway, amino acid metabolism, and fatty acid metabolism.

A significant current research focus is the metabolic reprogramming of immune cells, particularly macrophages. These cells, originating from various phagocyte lineages and expressing numerous immune receptors, are vital for pattern recognition. In the lungs, pulmonary macrophages act as the first line of defense against airborne particles and microbes, playing a critical role in maintaining immune homeostasis within the pulmonary system (62). The functional diversity of macrophages is driven by their polarized phenotypes, which are closely linked to significant metabolic changes, a phenomenon often referred to as immunometabolism (63). Proinflammatory macrophage polarization is associated with increased glycolysis, the pentose phosphate pathway, and fatty acid synthesis, while anti-inflammatory macrophages predominantly rely on OXPHOS, glutamine metabolism, and FAO (64). These metabolic adaptations are essential for maintaining macrophage function and polarization within specific physiological and pathological contexts.

#### 4.2.5 Targeting metabolic reprogramming to treat lung diseases

Metabolites play multifaceted roles in organisms, far beyond their function as simple markers of phenotypic traits. They have the ability to bind tightly to proteins, thereby regulating protein functions and influencing disease-related signal transduction pathways, which is crucial to the progression of various diseases. In modern scientific research, uncovering the relationships between metabolites and their direct target proteins, as well as elucidating how these interactions affect metabolic function, has become a key measure of research depth and innovation. In the context of lung diseases, alterations in metabolic pathways are increasingly recognized as significant biomarkers and therapeutic targets. Specifically, pathways involved in glucose, fatty acid, and amino acid metabolism play pivotal roles in the onset and progression of lung diseases. Enhanced glycolysis, for example, is a hallmark of several conditions, including COPD and pulmonary fibrosis, where cells rely on aerobic glycolysis even in oxygen-rich environments. This metabolic shift not only sustains cell proliferation but may also exacerbate inflammation. Furthermore, FAO is critical for maintaining alveolar epithelial cell function and mitigating oxidative stress, while glutamine metabolism supports cell proliferation and antioxidant defenses. Studying these metabolic pathways deepens our understanding of lung disease mechanisms and provides insight into identifying novel therapeutic targets. Potential targets include key metabolic enzymes and signaling pathways such as lactate dehydrogenase, pyruvate kinase, mTOR, and AMPK. Therapeutic interventions aimed at these targets could modify the metabolic state of cells, reduce inflammation, and slow disease progression. Additionally, exploring new therapeutic strategies that incorporate metabolic modulation opens up promising directions for lung disease treatment. For instance, drugs that modulate metabolism, such as metformin, or approaches that combine metabolism-focused treatments with immunotherapy, may improve therapeutic outcomes. Moreover, personalized treatment based on patients’ unique metabolic profiles can further optimize therapeutic effectiveness and minimize side effects.

In summary, in-depth exploration of metabolic mechanisms and the identification of new therapeutic targets in lung diseases, along with the refinement of existing treatment regimens from a metabolic perspective, offer innovative strategies for clinical management in this field.

Metabolic reprogramming is a fundamental cause and pathophysiological basis of lung disorders, including COPD. Disruptions in cellular function, manifesting as airway inflammation and remodeling, have been linked to aberrations in key energy metabolism pathways such as glycolysis and OXPHOS. The restoration of OXPHOS has shown therapeutic benefits in several respiratory diseases, including COPD, indicating that targeting metabolic pathways could be a promising treatment approach. Conversely, inhibiting glycolysis has proven effective in managing diseases like COPD. However, the precise regulation of OXPHOS and its related catabolic pathways—including mitochondrial pyruvate catabolism, FAO, and glutaminolysis—remains unclear in the context of these respiratory conditions.

The expanding range of metabolic enzymes connected to established metabolic pathways presents promising opportunities for treating respiratory illnesses. Further research is essential to fully understand the role of OXPHOS in various respiratory conditions, under different stimuli, and across diverse cell populations. Metabolic reprogramming is a pervasive feature of respiratory disorders, and while the efficacy of glycolysis inhibition is well-established in asthma, IPF, and COPD, the contribution of OXPHOS in these diseases remains incompletely understood.

Future research should focus on characterizing the key cellular changes involved in the dysregulation of OXPHOS in lung diseases like COPD. Given the complex adaptability of metabolic pathways that utilize OXPHOS across various cell types and microenvironments, identifying these dysregulated pathways could unveil potential therapeutic targets for treating respiratory diseases through metabolic reprogramming.

Several drugs targeting various aspects of cell metabolism are currently in clinical development for the treatment of multiple diseases (65). However, caution is necessary when manipulating metabolic pathways, as inhibiting these processes could negatively impact normal pulmonary immune responses. Such inhibition may reduce cellular energy production, biosynthetic capacity, and ROS generation, all of which are essential for mounting appropriate responses to inhaled pathogens. Recent advances in immune metabolism, especially within the innate immune system, have been notable. Modulating immune metabolism to influence macrophage polarization represents a promising strategy for managing lung disorders. This approach extends beyond macrophages to include other immune cells such as T cells, B cells, and NK cells, with implications for both neoplastic conditions and diseases characterized by immune dysregulation. Investigating the immunometabolism of these cell types in future studies could provide significant insights into the pathophysiology of lung diseases. The exploration of metabolic reprogramming sheds light on the regulatory processes governing immune cell function and can inform the development of innovative therapies aimed at immune modulation.

Multiple studies, particularly those using animal models, have demonstrated that metabolic reprogramming plays a vital role in maintaining innate immune homeostasis. However, due to the limitations of animal studies in accurately replicating the human *in vivo* environment, there is an increasing need to explore how metabolic pathways are disrupted in the immune cells of individuals with lung diseases. Such investigations are critical for identifying new biomarkers for disease endotyping and uncovering potential therapeutic targets. This area of research is particularly significant given the numerous metabolic pathways that are already pharmacologically targetable, with several inhibitors undergoing clinical trials for the treatment of conditions like cancer and pulmonary hypertension.

### 4.3 Comprehensive correlation literature metrology research and results

The inaugural study in this field, published in 1993, examined naphthalene, a common environmental contaminant known for its selective pulmonary toxicity in mice, and potentially implicated in human lung disease. This research demonstrated that measuring urinary metabolites of naphthalene provides a noninvasive method to assess toxic exposure. Following this breakthrough, the field gained traction, and in the 2000s, researchers from various countries began investigating the link between metabolism and COPD. Initial studies, though few in number, focused on identifying metabolic alterations in patients with COPD. By 2010, the scope had expanded to include other lung diseases, with increasing attention paid to uncovering the molecular mechanisms associated with these metabolic changes. The volume of research grew substantially, attracting a larger scientific community. Since 2015, the focus has shifted toward examining how different drugs can influence disease progression in lung disorders, particularly by exploring their mechanisms of action. Lung metabolism has garnered significant attention due to its profound impact on human health. Current research continues to explore the interaction between metabolism and lung diseases, with a strong emphasis on drug discovery aimed at modulating lung metabolism. This field is expected to advance further as more researchers delve into these mechanisms. The collaborative network in this area spans coauthor relationships across countries and institutions. By leveraging clustering from co-cited reference networks, researchers can visually map the contributions of different teams to scientific progress and identify potential collaborators. Examining the roles of nations and institutions in this network may also facilitate securing funding for key initiatives and fostering global partnerships.

Bibliometric analysis offers multiple advantages to researchers (66). It enables the extraction and visualization of articles, highlighting prevailing research themes tied to specific keywords. Additionally, identifying co-occurring author keywords and keyword burst networks helps pinpoint relevant terms for refining database searches. This analysis also aids in tracking evolving research trends, identifying emerging areas of interest, and understanding productivity patterns and pivotal moments in knowledge development. Seminal papers, which serve as foundational works within a research cluster, are critical for understanding the trajectory of the field. Moreover, journal and co-citation analyses provide strategic insights for selecting the most suitable journals for publication.

### 4.4 Limitations and strengths

Recognizing certain inherent limitations is essential, particularly the reliance on citation-based indicators in bibliometric research, which can introduce biases, notably citation bias (67). This constraint stems from the exclusive use of the WOS SCI-E database, excluding other resources like PubMed and Scopus. Despite this, the selected publications are considered to offer a comprehensive representation of the field. Additionally, the reference data from WoS are highly accurate, consistent, and free from redundancy, covering essential details such as titles, authors, and institutions for bibliometric analysis. WoSCC remains the leading database used in bibliometric studies (12). The search terms applied—TS = (“metabolism”) and TS = (“lung disease” OR “pulmonary disease”)—might not capture the full scope of literature related to the interplay between lung disease and metabolism. Despite these constraints, the study offers valuable insights into this relationship and provides a solid foundation for future research efforts.

To mitigate these limitations, future research should employ a broader and more diverse set of keywords to encompass relevant synonyms and variations, potentially providing a more comprehensive perspective. Despite these constraints, this study offers valuable insights into the relationship between lung disease and metabolism, laying a strong foundation for subsequent research. The literature search began in 1991, which served as the starting point for this investigation. Articles published before 1990 may have been excluded due to limitations in electronic records, but this temporal constraint had a minimal impact on the results. Another concern is that citing a large number of review articles may introduce bias in citation-based analyses. As is common in bibliometric studies, review articles were excluded from the analysis to minimize this effect. Unlike previous reviews, which are often limited in content and references, this study provides a more dynamic view, capturing the evolution of the field and offering a comprehensive understanding of metabolism in lung diseases. This analysis deciphers influential research, maps disparate topics, and tracks emerging trends. Notably, other lung diseases linked to metabolism—such as lung cancer, IPF, infectious lung diseases, and asthma—did not appear as prominent keywords in the co-occurrence and burst analysis shown in Figure 6A. This absence does not imply a lack of connection to metabolism. However, our focus is on two disease types with significant co-occurrence and burst keywords, which are explored in detail in the discussion section. This study represents a pioneering effort in conducting a bibliometric analysis of metabolism in lung diseases, setting it apart from traditional narrative reviews. It offers an in-depth examination of research trends and historical developments, benefiting clinicians and scholars alike. Furthermore, this research has the potential to highlight unexplored areas for future clinical trials that address key questions in the field. It also helps identify leading authors and reputable journals focused on the role of metabolism in lung diseases, providing valuable guidance for early-career researchers in selecting mentors and institutions and aligning their research goals with the priorities of stakeholders, policymakers, and funding bodies within the clinical and scientific communities.

## 5 Conclusion

Metabolic dysregulation in lung cells has been increasingly recognized as a key factor in the pathogenesis of various pulmonary diseases. The effects of altered energy metabolism on the pathophysiology of these conditions have become a growing area of research, though many questions remain unresolved. Further investigation into cellular energy metabolism is expected to uncover new therapeutic strategies for lung diseases. The understanding of how metabolism influences the progression of these diseases has been significantly advanced by the thorough examination of scholarly work by previous researchers. Using CiteSpace, this study was able to synthesize key molecular mechanisms and focal research areas, providing deeper insights into the complex relationship between metabolism and lung disease development.

## Data Availability

The original contributions presented in the study are included in the article/supplementary material, further inquiries can be directed to the corresponding author.

## Footnotes

^1^ http://clarivate.com/products/web-of-science

^2^ http://bibliometric.com/

^3^ https://genemania.org/
